# RNA based viral silencing suppression in plant pararetroviruses

**DOI:** 10.3389/fpls.2015.00398

**Published:** 2015-06-10

**Authors:** Thomas Hohn

**Affiliations:** ^1^Botanical Institute, University of BaselBasel, Switzerland; ^2^Friedrich Miescher InstituteBasel, Switzerland

**Keywords:** caulimovirus, silencing, silencing suppression, translation, shunting, POL II, viroid, siRNA

## Abstract

The 35S promoter of cauliflower mosaic virus and that of other plant pararetroviruses gives rise to an RNA, which is both a pre-genome and a polycistronic mRNA. The 600 nucleotide long very structured leader of this RNA is also transcribed separately. The resulting 8S RNA is then converted to a double strand giving rise to a huge set of siRNAs, which suppress silencing. In this Mini-Review I discuss how this versatile stretch of 600 nts constitutes a masterpiece of evolution.

## Introduction

Plants respond to virus infections mainly by RNA silencing (RS). RS is generally initiated by recognition of double stranded RNA, usually accumulating as a by product of virus replication. In addition for some cases effector-triggered immunity (ETI) to virus infections was reported (Table 1 in Zvereva and Pooggin, 2012), ETI is initiated by an interaction of viral effectors with intracellular NB–LRR proteins and leads in most cases to hypersensitive response (HR), death of the infected cells and systemic acquired resistance (SAR); Successful virus infections depend on viral counter actions mediated by suppressors (VSRs) interfering with silencing (Szittya and Burgyán, 2013) and at least in some cases on viral avirulence proteins (Avrs) blocking ETI (Zvereva and Pooggin, 2012).

Silencing is initiated by transcription of virus RNAs by viral or host RNA-dependent RNA polymerases (RDRs) to yield dsRNAs. These are cleaved by dicer-like proteins (DCLs) into 21–24 nt small RNA duplexes (siRNAs). *Arabidopsis thaliana* has four dicers. The ds RNAs derived from cytoplasmic RNA viruses are diced by DCLs 4 and 2, while those derived from viruses establishing minichromosomes, i.e., geminiviruses and caulimoviruses, are cleaved in addition by DCLs 1 and 3 (Akbergenov et al., 2006; Blevins et al., 2006; Moissiard and Voinnet, 2006). The siRNA duplexes are stabilized by methylation of the 2′OH groups at their 3′-termini. The duplexes are melted and the single-strand “guide-siRNAs” are picked up by Argonaute proteins (AGOs) to form RNA-induced silencing complexes (RISCs). These are guided to cognate virus RNA strands, where they induce RNA cleavage or inhibition of translation (Brodersen and Voinnet, 2006; Hohn and Vazquez, 2011; Pikaard and Mittelsten-Scheid, 2014).

Individual viruses use different, usually unrelated viral proteins to interfere with silencing by binding to dsRNA, inhibiting or degrading dicers, interfering with or inactivating AGO proteins or interacting with loaded RISCs (Szittya and Burgyán, 2013). Since viral RNAs are targets rather than inhibitors of silencing they have not as yet been considered as silencing suppressors. However, recent data obtained with plant pararetroviruses, such as *Cauliflower Mosaic Virus* (CaMV), and *Rice Tungro Bacilliform Virus* (RTBV) allow the extension of the list of viral suppressors to ds viral suppressor RNAs (Blevins et al., 2011; Rajeswaran et al., 2014a).

## *Cauliflower Mosaic Virus*, a Short Warrant

**FIGURE 1:**
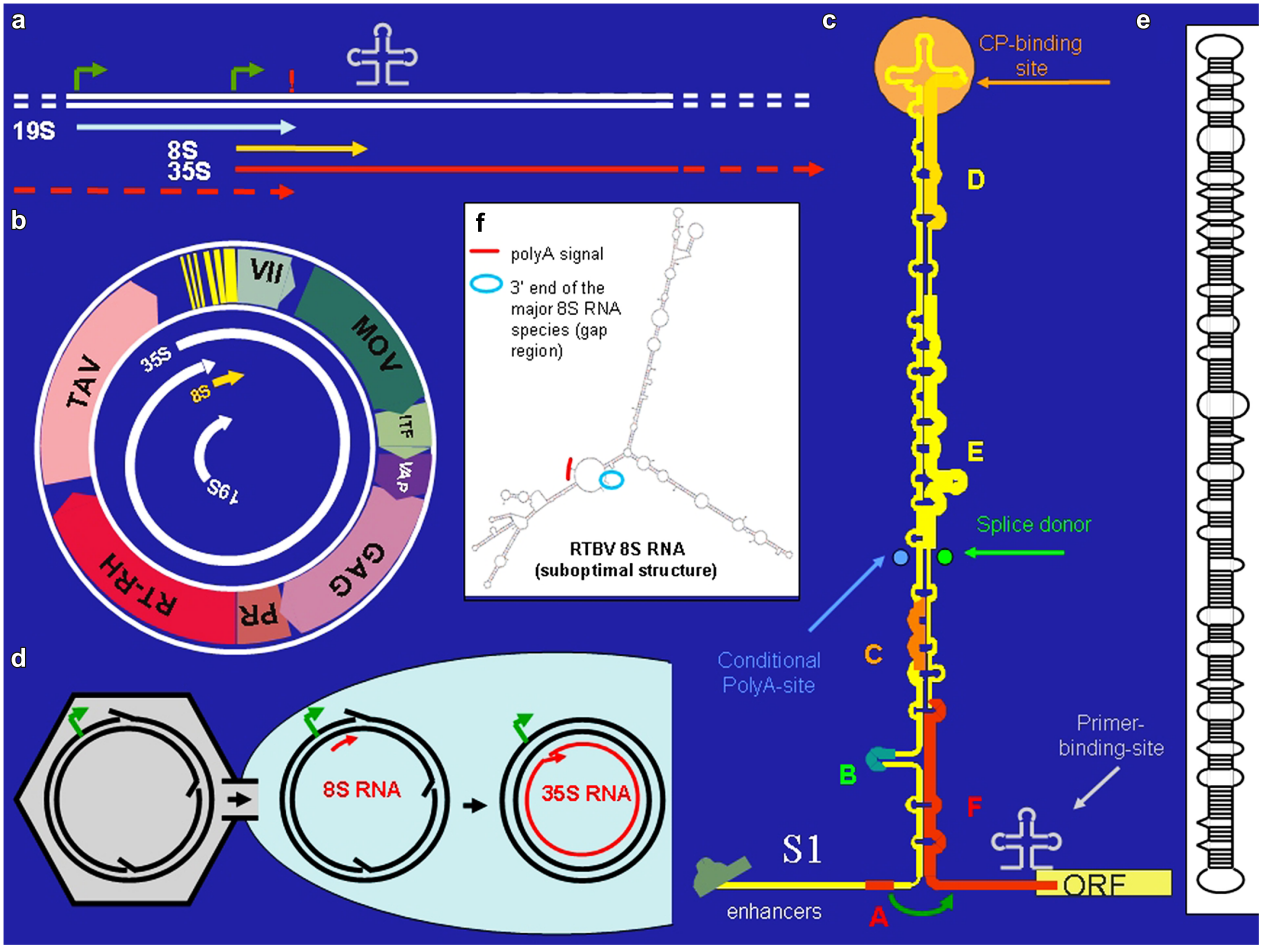
***Cauliflower Mosaic Virus* (CaMV). (a)** Positions of promoters (bent arrows), primer binding site (cloverleaf), polyadenylation signal (!) on the CaMV DNA, and the positions of the CaMV individual CaMV RNAs. **(b)** The 8 kb long circular CaMV DNA, its transcripts and its coding regions [ORF VII, no obvious function; MOV, movement protein; ITF, insect transmission factor; VAP, virion associated protein; GAG, capsid protein, PR/RT-RH, protease and reverse transcriptase fused coding region (POL); TAV, transactivator/viroplasmin]. **(c)** The CaMV RNA leader with its compact secondary structure. Capital letters correspond to small ORFs. Special features are indicated. The roundish arrow symbolizes the shunt process. **(d)** Nuclear entry of open circular CaMV DNA. **(e)** Structure of pospiviroid. **(f)** Suboptimal folding of *Rice Tungro Bacilliform Virus* (RTBV) 8S RNA revealing spatial vicinity of the facultative polyadenylation signal and the fall-off site.

*Cauliflower Mosaic Virus* is a plant pararetrovirus, the genome of which accumulates in the infected plant nucleus in multiple copies of an 8 kb circular minichromosome. Within virus particles the DNA circle is relaxed due to three short gaps with overhangs (**Figure 1d**) that mark the starts/ends of minus- and plus-strand DNA synthesis. The minus strand gap is located at the primer (met-tRNA) binding site, the other two at polypurine stretches. (For reverse transcription the met-tRNA primes minus strand DNA synthesis, while the polypurine stretches prime plus strand DNA synthesis).

*Cauliflower Mosaic Virus* encodes seven proteins (**Figure 1b**). Of special interest is the unique transactivator/viroplasmin (TAV). TAV is a multifunctional protein forming viral inclusion bodies and enabling polycistronic translation and virus assembly (reviewed in Hohn and Rothnie, 2013). TAV also acts as elicitor of innate immunity (Love et al., 2012; Zvereva and Pooggin, 2012) and as silencing suppressor, inhibiting the RDR6/DCL4-dependent 21 nt siRNA pathway (Haas et al., 2008; Shivaprasad et al., 2008; Hohn, 2013).

*Cauliflower Mosaic Virus* produces three primary RNAs: 35S RNA, 19S RNA, and 8S RNA (Guilley et al. (1982; **Figure 1a**). The 35S RNA covers the whole genome and is terminally redundant due to a conditional polyadenylation signal, which is passed at the first encounter with the transcription machinery, but recognized at the second (Sanfaçon and Hohn, 1990). It acts both as pregenomic and as polycistronic mRNA (Fütterer et al., 1988). Its translation depends on TAV, which is encoded by the subgenomic 19S RNA. The 8S RNA is non-coding. It coincides with the 600 nt long highly structured leader of the 35S RNA. Translation initiation from the 35S RNA depends on “shunting,” whereby the scanning ribosome bypasses the highly structured central portion of the leader (Hohn et al., 2002). Small open reading frame “A” in front of the central stem structure is required for this process (**Figure 1c**).

Complex and long leaders are not unique to CaMV. Inspection of 14 related pararetroviruses, including rod-shaped *Banana Streak Virus* (BSV) and RTBV (Pooggin et al., 1999) revealed that they all have comparable leaders with structural, but not sequence similarities. Like for CaMV, these carry several sORFs, the first of which is 5–10 nts away from the central stem structure and spatially close to the first true ORF, predicting a shunting mechanism similar to the one for CaMV. Shunting was explicitly shown also for RTBV (Pooggin et al., 2006, 2008).

**FIGURE 2:**
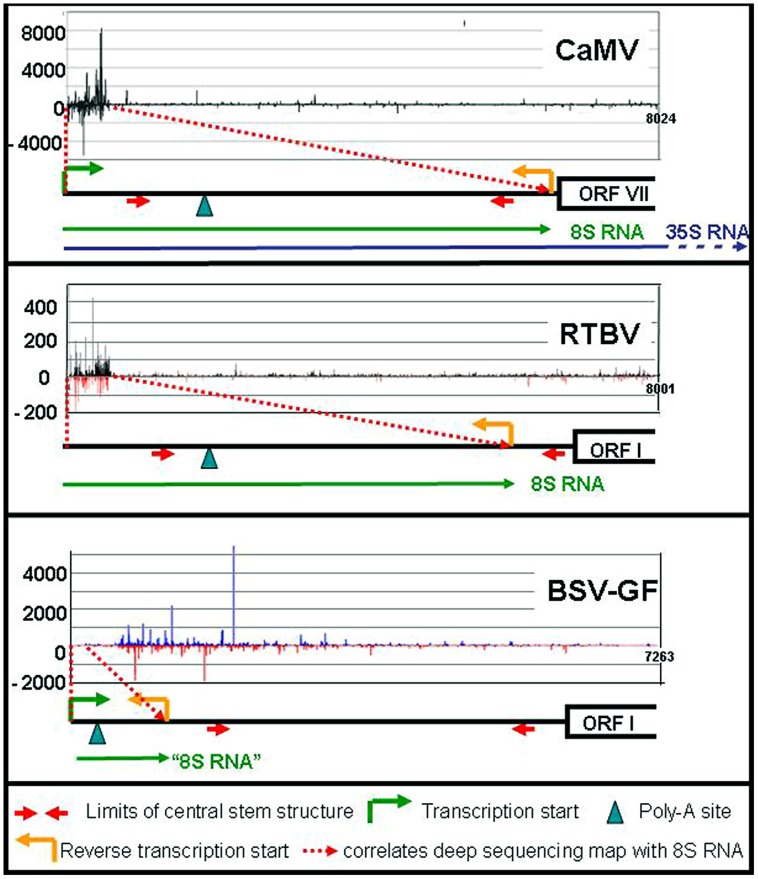
**siRNA mapping along caulimoviruses**. For each virus the relative amounts of its si-RNAs (black marks for CaMV, black, and red marks for RTBV, blue and red marks for BSV-GF) are plotted against the whole genome (Blevins et al., 2011; Rajeswaran et al., 2014a,b). The respective leader regions are presented below as black lines. The 8S RNAs of CaMV and RTBV are shown as green lines, as well as the corresponding 100 nt RNA of BSV (“8S RNA”). The 35S RNA is shown only for CaMV (blue line).

In addition to these major RNAs, all size classes of CaMV-derived siRNA (21–24 nts) of both polarities have been reported, making up half of the total amount of siRNAs in the infected plants. All four *Arabidopsis* DCLs including DCL1 are implicated (Blevins et al., 2006; Moissiard and Voinnet, 2006). Deep sequencing revealed that the bulk of those siRNAs are derived from the 600 nts of the 35S RNA leader region/8S RNA (82%), while siRNAs derived from the remaining 7400 nts of CaMV RNAs are rare (18%; **Figure 2**). DCLs 3, 1, and 2 are involved giving rise to species of 24 nt (47%), 21 nt (27%), and 22 nt (14%). Interestingly, the production of 21 nt long siRNAs by RDR6/DCL4/DRB4 is inhibited by TAV in its function as proteinaceous silencing suppressor (Haas et al., 2008; Shivaprasad et al., 2008).

## The 8S RNA

To learn more about the preferential siRNA production, 8S RNA was isolated and characterized in detail by circularization-reverse-transcription PCR (Blevins et al., 2011). It starts at exactly the same position as the 35S RNA (**Figure 1a**), has a cap and ends at a narrow cluster of positions close to the start/end of reverse transcription and lacks a poly-A tail. Interestingly, not only sense 8S RNA (s-8S RNA) was found, but also antisense 8S RNA (as-8S RNA). The as-8S RNA starts roughly where the s-8S RNA ends and ends exactly where the s-8S RNA starts. The as-8S RNA has neither a cap nor a poly-A tail.

How is the poly-A tail-less s-8S RNA produced? Cauliflower mosaic virions are guided to nuclear pores via nuclear localization signals (Leclerc et al., 1999). Due to their large size, the virions cannot enter the nucleus, but just deliver the open circular DNA (**Figure 1d**). There must be a time window until the gaps/overhangs of nascent CaMV DNA are removed by repair enzymes and ligase and the supercoil closed. If transcription is initiated before DNA closure, the nascent RNA may fall off at the gap/overhang of the DNA minus strand or near of it as s-8S RNA. A fall-off would explain the lack of polyadenylation. The length of s-8S RNA is thus defined by the distance between start of transcription from the 35S promoter and roughly the primer binding site.

The mechanism of as-8S RNA production is not yet known. At the relevant antisense positions CaMV DNA contains neither promoter-like sequences nor polyadenylation signals, making ordinary DNA-depending antisense transcription unlikely. Although promoters lacking TATA-boxes exist in plants and other organisms (Morton et al., 2014), transcription directed by them would still produce capped, and polyadenylated transcripts. Any type of transcription using the met-tRNA as a primer is also unlikely, since its sequence or part of it is not included in as-8S RNA. Furthermore, synthesis of the as-8S RNA requires neither RNA-dependent RNA-polymerases 1, 2, or 6 nor POL IV and POL V, ruling out their involvement (Blevins et al., 2006).

A possibility would be that a DNA-dependent RNA-polymerases (POL I, II, or III) is involved (Bonfiglioli et al., 1996 for POL I; Lehmann et al., 2007 for POL II). In fact, RNA dependent RNA-polymerase activity of Pol II has been observed by several authors (Wagner et al., 2013 and references therein). For instance, pospiviroid- and *Hepatitis Delta Virus* RNAs are replicated by POL II in an α-amanitin-sensitive mode and pospiviroids apparently make use of an RNA-based promoter located on highly structured circular viroid RNA (Pelchat et al., 2002; Flores et al., 2011). Inspection of the secondary structure of s-8S RNA (Fütterer et al., 1988) reveals an interesting resemblance to viroid RNA (**Figure 1e**): both RNAs have long stretches of imperfectly matched dsRNA. This suggests that also the ds form of 8S RNA might originate from transcription of 8S RNA by POL II, perhaps using an RNA-based promoter, as in pospiviroids (Bojić et al., 2012). Future experiments will be required to test this hypothesis.

Whatever the mechanism, the as-8S RNA production on the s-8S RNA template may either lead directly to an 8S-RNA duplex or the two strands may anneal later. A nuclear involvement of POL II in as-8S RNA production and duplex formation would be in line with the high proportion of 24 nt long siRNAs produced by the nuclear dicer DCL 3 (see below).

## 8S-RNA Derived siRNAs and the Decoy Model

One very attractive hypothesis suggests a function for the siRNAs derived from the 8S RNA duplex: they may act as decoys competing with the remaining siRNAs for free AGO proteins. This would also explain the very low amount of siRNAs produced from the CaMV coding region. Experiments using AGO1 antibodies indeed showed that 21 and 22 nt long siRNAs derived from the 8S RNA were bound to AGO1, while those derived from the remaining CaMV region did not Blevins et al. (2011).

The 8S derived siRNAs on the other hand cannot efficiently target the 8S RNA itself or the leader of 35S RNA. The compact structure of these RNA sequences renders them as unfavorable targets for AGO-RISCs. A similar effect is discussed for viroids, the rod-like structures of which are perfect targets for DCLs but very poor ones for AGO/RISCs (Itaya et al., 2007; Pumplin and Voinnet, 2013).

If the decoy model is correct, the 8S RNA should also lead to large amounts of siRNAs in a chimeric context. To test this, the CaMV 8S RNA was ectopically expressed in a *Cabbage Leaf Curl (Gemini) Virus* (CaLCuV) vector, leading to s-8S like RNA, in this case in a polyadenylated version. Also in this connection large amounts of 8S RNA-derived siRNAs of both polarities were observed, the majority of which was again 24 nt long. The chimeric virus was produced in higher amounts than the empty vector or a CaLCuV vector loaded with a GUS gene, again indicating RNA-based silencing suppression (Blevins et al., 2011).

On the other hand, no substantial general reduction of **host** small RNAs was observed during CaMV infection. This might have to do with compartmentalization, separating virus, and host siRNAs spatially. For instance, 24 nt long siRNAs together with POL IV accumulate in Cajal bodies inside the nucleolus (Li et al., 2006; Pontes et al., 2006), while the viral ones might accumulate outside the Cajal bodies. miRNAs might evade suppression by successfully competing for AGO1 with siRNAs; including those derived from the 8S RNA.

## Shunting and Decoy, Comparison with other Plant Pararetroviruses

RNA-based silencing suppression might be a general strategy of plant pararetroviruses. Sense and antisense 8S RNAs were also identified in RTBV infected rice plants (Rajeswaran et al., 2014a). In this case the s-8S RNA was more precisely terminated at the corresponding gap/overlap of the minus DNA strand than the 8S RNA of CaMV. Interestingly a minority of the RTBV s-8S RNAs had a short poly-A tail. Although no poly-A signal was found close to this polyadenylation site, inspection of an, albeit suboptimal RTBV 8S RNA secondary structure reveals a spatial neighborhood between the facultative polyadenylation signal and the fall-off site (**Figure 1f**). This resembles the case of *human T-cell leukemia virus* (HTLV), where a polyadenylation signal is moved over a distance of 290 nts to the facultative polyadenylation site through secondary structure (Shimotohno et al., 1984).

Like in the case of CaMV 8S RNA, huge amounts of siRNAs are produced also from the RTBV-derived 8S-RNA duplexes (Rajeswaran et al., 2014a). Since RTBV is phloem-limited, naturally their percentage is lower (17% compared to 83% host sRNAs). Again, the majority of the 8S-derived siRNAs are 24 nt long and very few siRNAs were derived from the RTBV coding region (**Figure 2**).

Different results were obtained for BSV-GF (Rajeswaran et al., 2014b). For BSV-GF and most other BSV isolates only a very short stretch (∼100 nts) of RNA is located between promoter and fall-off site, apparently too short for efficient asRNA and siRNA production (**Figure 2**).

Comparison of the three viruses confirms that 8S RNAs are produced by fall-off at the primer binding site, if properly spaced, and that it does not depend on the compact structure of the leader, which is present in all these three viruses. This compact structure of the leader, however, protects it from AGO-RISC-dependent degradation.

## Conclusion 1: A Masterpiece of Evolution

The stretch of 600 nts comprising both, the leader of the CaMV pregenomic RNA and the blunt-ended 8S RNA constitutes a masterpiece of evolution. Due to its position it allows unusual fall-off transcription, due to its compact secondary structure it is resistant to AGO-mediated degradation. This secondary structure apparently leads also to an unusual replication mechanism, giving rise to antisense 8S RNA, which hybridizes with its template yielding 8S RNA duplexes as source of huge amounts of decoy siRNAs. The obvious disadvantage of such a structure: inhibition of ribosome scanning and translation is compensated by an ingenious positioning of a small open reading frame, which initiates a shunt mechanism leading the scanning ribosome directly to the start site of translation.

## Conclusion 2: Implications

Analysis of the siRNA patterns in pararetrovirus-infected plants have led to the discovery of a novel silencing suppression strategy. Although mechanistic details await further experimentation, at least some players of the game are testable. Future science will reveal whether RNA-based silencing suppression is a more widely used strategy and whether host organisms have developed strategies to fight such activity.

## Conflict of Interest Statement

The author declares that the research was conducted in the absence of any commercial or financial relationships that could be construed as a potential conflict of interest.
